# Rapamycin increases the incidence of neuropsychiatric illness in kidney transplant patients through the suppression of neural stem cells

**DOI:** 10.1038/s41398-020-0838-2

**Published:** 2020-05-18

**Authors:** Yangsik Kim, Jung Sun Lee, Yeon Ho Joo

**Affiliations:** 1 Department of Psychiatry, Asan Medical Center, University of Ulsan College of Medicine, Seoul, South Korea; 2 Graduate School of Medical Science and Engineering, Korea Advanced Institute of Science and Technology, Daejeon, South Korea; 3 Center for Synaptic Brain Dysfunction, Institute for Basic Science, Daejeon, South Korea; 4 Mental Health Research Institute, National Center for Mental Health, Seoul, South Korea

**Keywords:** Molecular neuroscience, Stem cells, Depression

## Abstract

Rapamycin inhibits protein translation in cells, including neural stem cells (NSCs), by suppressing the mechanistic target of rapamycin (mTOR). This drug has been widely used together with calcineurin inhibitors in transplantation patients to prevent graft rejection. Previous studies have reported an association between mTOR and depression, but few investigations of this have occurred in transplant recipients. We have here tested the psychiatric effects of rapamycin in mice. The animals treated with rapamycin showed decreased locomotion and sugar consumption. In these rapamycin-treated mice also, the granule cells in the dentate gyrus (DG), which actively differentiate and proliferate from NSC, showed decreases in both excitatory and inhibitory synaptic transmission. Furthermore, the SOX2/NeuN ratio in the DG was decreased in mice treated with rapamycin. We further show that kidney transplantation patients who are receiving rapamycin have more psychiatric disorder such as adjustment disorder. Clinical attention is thus needed when administering rapamycin to transplant recipients due to its behavioral effects and its impact on NSC.

## Introduction

Kidney transplantation (KT) is an effective intervention for end-stage renal disease, as the patient no longer requires time-consuming hemodialysis and it prevents chronic renal failure (CRF). However, KT recipients must take immunosuppressants to prevent graft rejection. The calcineurin inhibitors cyclosporine and tacrolimus, and the mechanistic target of rapamycin (mTOR) inhibitors rapamycin, sirolimus, and everolimus, are commonly used for maintenance treatment in KT patients to suppress immune responses to the allograft kidney^1,2^.

mTOR is a central modulator of the initiation of protein translation^3^ and is activated by Akt, also known as protein kinase B, from the phosphoinositide-3 kinase signal cascade. mTOR is also regulated by the energy state of the body. Nutrients such as amino acids promote mTOR activation, but an energy-deprived state inhibits mTOR. In neurodevelopment, mTOR induces neural stem cell (NSC) differentiation and proliferation^4,5^. Prior genetic studies have found that the hyperactivation of mTOR caused by its upstream modulator phosphatase and tensin homolog (PTEN) deletion and a TSC1 deletion increases the NSC size and number, and augments dendritic spine arborization, synaptic branching, and synaptic excitation^6,7^. Aberrant activation of mTORC1 caused by a TSC2 deletion presents as a loss of stemness. The neuropsychiatric disorders associated with mTOR hyperactivation are related to neurodevelopmental disorders such as epilepsy and autism spectrum disorder^8^.

Previous postmortem studies in patients with depression have uncovered deficits in the *N*-methyl-d-aspartate receptor (NMDAR) and mTOR signaling in the prefrontal cortex^9,10^. In addition, many prior studies have reported that the NMDA antagonist ketamine has an antidepressant effect through mTOR activation^11–19^.

In our current study, we assessed the effects of rapamycin on psychiatric illness in mouse models using behavioral, electrophysiological, and immunofluorescence methodologies. We further investigated the prevalence of psychiatric disorders and psychiatric drug prescription use in KT patients who had been receiving rapamycin or not. Our findings indicate that rapamycin causes behavior and electrophysiological changes, and has negative effects on the proliferation of NSC.

## Materials and methods

### Animal studies

B6J male mice were provided with free access to food and water and three to four animals were housed per cage under a 12 h light–dark cycle. All mice were bred and maintained in accordance with the requirements of the Animal Research at Korean Advanced Institute of Science and Technology (KAIST), and all procedures and methods were approved by the Committee of Animal Research at KAIST (KA201). Mice were randomly assigned to a rapamycin or control vehicle group. Eight-week-old mice in each group were administered rapamycin or vehicle solution for 4 weeks, respectively. The rapamycin and vehicle solutions were prepared as follows: 6.75 µl/ml concentration of 25 µg/ml rapamycin/dimethyl sulfoxide (DMSO) in 5% w/u PEG8000 in water (rapamycin, LC Laboratory, MA; DMSO and PEG8000, Sigma, MI). At 2 weeks after the ingestion of these solutions, behavioral tasks were given to the mice. At 4 weeks after ingestion, electrophysiology assessments using patch-clamp, immunofluorescence, and protein level analyses using immunoblotting were conducted. All mouse experiments were repeated with two cohorts.

### Patient studies

This study was approved by Institutional Review Board of Asan Medical Center (2019–0332). We reviewed the electronic medical records of our study patients using ABLE (Asan Biomedical Research Environment) system, an Structured Query Language-based database. We enrolled patients who underwent KT between 1 January 2009 and 31 December 2018 (Supplementary Fig. 1). Patients were classified according to rapamycin use. All of the KT patients had also been prescribed a calcineurin inhibitor. We screened the study patient medical records using the ICD-10 code for the diagnosis of psychiatric disorders including depression (major depressive disorder (MDD), depressive episode, dysthymia), anxiety (anxiety disorder not otherwise specified, generalized anxiety disorder, panic disorder, specific phobia, obsessive-compulsive disorder), delirium, somatoform disorder, insomnia, opioid-use disorder (opioid abuse, opioid dependence, opioid withdrawal), and psychosis; diabetes mellitus (DM) including type 1 and type 2 DM; and primary kidney disorders including chronic kidney disease, glomerular disease, renal tubero-interstitial disease, and polycystic kidney disease. Psychiatric medication use among these subjects was also investigated including antidepressants (selective serotonin reuptake inhibitor, selectivie serotonin and norepinephrine reuptake inhibitor, bupropion, and mirtazapine), antipsychotics (clozapine, olanzapine, risperidone, paliperidone, aripiprazole, amisulpride, ziprasidone, haloperidol, and chlorpromazine), benzodiazepine (lorazepam, diazepam, alprazolam, clonazepam, bromazepam, and etizolam), and zolpidem. This study was approved by the institutional review boards at Asan Medical Center.

### Statistical analyses

Statistical data analysis was performed using R 3.5.1 and Prism 8 (GraphPad). Data normality was determined using a Shapiro–Wilk normality test. Data with a normal distribution were analyzed using the Student’s *t*-test and analysis of variance (ANOVA), followed by post-hoc tests. Data failing the normality test were analyzed using the Mann–Whitney test. The ROUT method was used to exclude outliers with a *Q*-coefficient of 1%. Specific numbers and statistical details are shown in Supplementary Table 1.

Further details on the materials and methods used in this study are provided in the Supplementary Information.

## Results

### Decreased locomotion and sugar consumption in mice treated with rapamycin

**Fig. 1:**
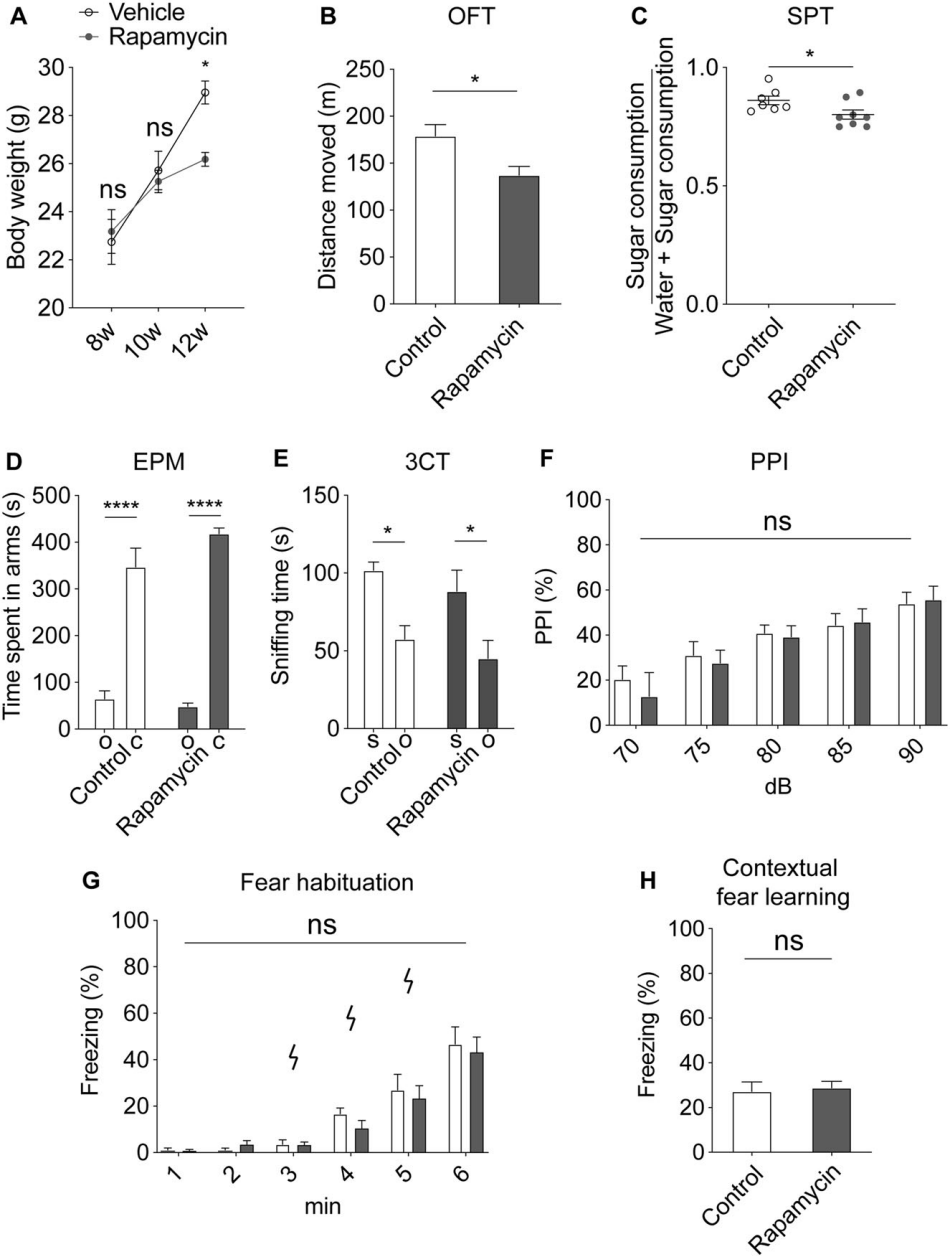
Mice treated with rapamycin shows depression-like behaviors. **a** After 4 weeks of treatment with rapamycin, the mice showed decreased body weights compared with the animals treated with control vehicle, *n* = rapamycin 8, control 7, two-way ANOVA. Bonferroni’s post hoc. **b** Mice treated with rapamycin showed decreased locomotion in an open-field test (OFT), *n* = rapamycin 8, control 7, Student’s *t*-test. **c** Mice treated with rapamycin had a decreased sugar consumption in a sugar preference test (SPT), *n* = rapamycin 8, control 7, Student’s *t*-test. **d** The rapamycin and control groups showed no difference in an elevated plus maze (EPM) test for anxiety behavior; O, open arm, C, closed arm; *n* = rapamycin 8, control 7, two-way ANOVA. **e** A three-chambered social interaction test (3CT) revealed no differences in social preferences between mice treated with rapamycin or vehicle; S, social target, O object. *n* = rapamycin 8, control 7, two-way ANOVA. **f** Sensory gating function measured by prepulse inhibition (PPI) was comparable between mice treated with rapamycin or vehicle, *n* = rapamycin 8, control 7, two-way ANOVA. **g**, **h** A fear learning test with foot shock was used to test for learning ability. Foot shocks were given at 3, 4, and 5 min. Fear habituation and contextual fear learning were comparable between mice treated with rapamycin or vehicle. Fear habituation, *n* = rapamycin 8, control 7, two-way ANOVA. Contextual fear learning, *n* = rapamycin 8, control 7; Student’s *t*-test; ns, not significant, *p < 0.05, ****p < 0.0001.

We used a mouse model to test the effects of rapamycin on specific behaviors^20^. The mice treated with rapamycin showed a decreased body weight compared with control vehicle-treated animals (Fig. 1a). Immunoblotting of the brain tissue from the rapamycin mouse group showed decreased levels of phospho-S6 kinase, a protein which is activated by mTOR, compared with the control animals (Supplementary Fig. 1). The mice treated with rapamycin also showed reduced locomotion and sugar consumption, which have been found to reflect depressive symptoms in humans (Fig. 1b, c). However, social behaviors measured using a three-chambered social interaction test, anxiety measured via an elevated plus maze, and sensory gating assessed with a prepulse inhibition appeared to be unaffected in the mice receiving rapamycin (Fig. 1d-f). Learning ability was also unaffected by rapamycin in these mouse experiments (Fig. 1g, h).

To examine the behavioral changes after rapamycin discontinuation in mice treated with rapamycin, behavioral experiments were conducted after discontinuation of rapamycin for 2 weeks. Rapamycin is a drug with a half-life of about 15 h in mice^21,22^ and, given the effect of rapamycin on translation, the drug was stopped for 2 weeks for a sufficient washout period. After stopping rapamycin for 2 weeks, mice still showed less weight compared with animals treated with the control vehicle (Supplementary Fig. 2A). Mice treated with rapamycin also showed decreased locomotion and sugar consumption compared with the control animals after rapamycin discontinuation (Supplementary Fig. 2B, C).

### Decreased excitatory and inhibitory transmission in the DG of mice treated with rapamycin

Proliferating cells are known to have more translational activity. Rapamycin initiates translation through mTOR, its downstream target activation of S6K, and via the inhibition of 4E-BP1^3,5^. The dentate gyrus (DG) is one of the most active regions of the brain in terms of cellular differentiation and proliferation. NSC in the DG differentiate into neural progenitor cells, which finally become mature granule cell neurons, and migrate from the hilum to the inner granular layer of the DG. Moreover, the DG is known as one of the areas most affected by depressive disorders^23,24^. We thus measure the electrophysiological properties of the neurons in the inner granular layer of the DG in the rapamycin-treated mice.

**Fig. 2:**
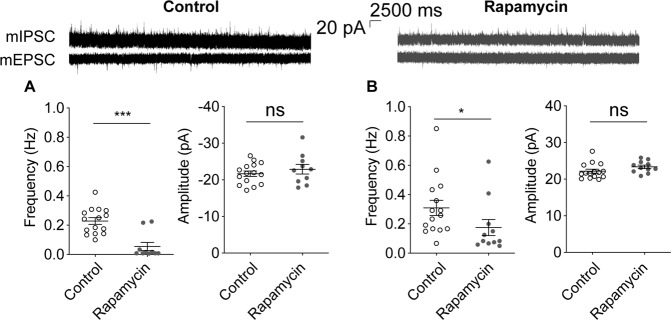
Excitatory and inhibitory synaptic transmission in the DG granule cells are decreased in mice treated with rapamycin. **a** The miniature excitatory synaptic current (mEPSC) frequency in the DG granule cells is decreased in mice treated with rapamycin compared with mice treated with vehicle. The mEPSC amplitude was not different between these groups however. mEPSC frequency, *n* = rapamycin 10 (3 mice), control 15 (3 mice), Mann–Whitney test; mEPSC amplitude, *n* = rapamycin 10 (3 mice), control 15 (3 mice), Student’s *t*-test. **b** The miniature inhibitory synaptic current (mIPSC) frequency in the DG granule cells is decreased in mice treated with rapamycin compared with mice treated with vehicle. *n* = mIPSC frequency, rapamycin 11 (3 mice), control 15 (3 mice), Mann–Whitney test; mIPSC amplitude, *n* = rapamycin 11 (3 mice), control 15 (3 mice). Student’s *t*-test, ns, not significant, *p < 0.05, ***p < 0.001.

The miniature excitatory synaptic transmission (mEPSC) frequency of the DG granule cells showed a profound decrease in the mice treated with rapamycin (Fig. 2a). The mEPSC amplitude was unaffected however. In addition, the miniature inhibitory synaptic transmission (mIPSC) frequency of these granule cells had a modest reduction in the mice treated with rapamycin without a change in amplitude (Fig. 2b). These results can be attributed to a decrease in synaptic numbers as a result of reduced synaptic growth and cell proliferation, which can explain the effects of rapamycin on synaptic plasticity.

### SOX2 is decreased in the DG of mice treated with rapamycin

**Fig. 3:**
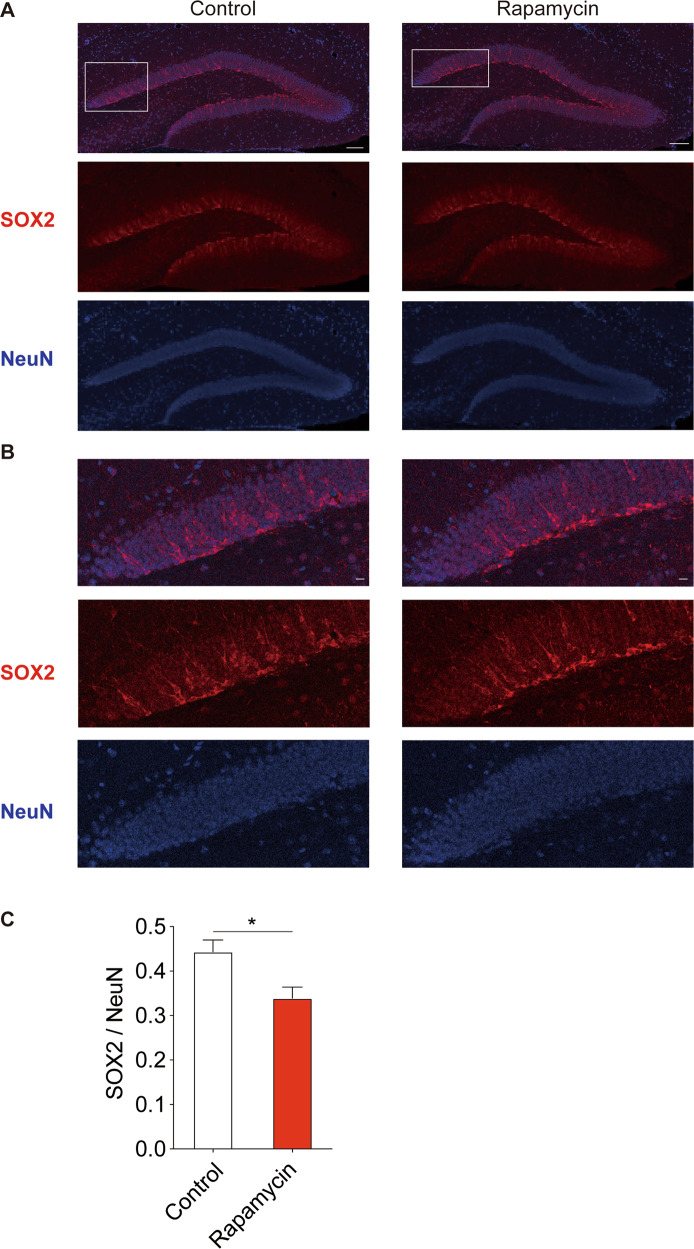
The SOX2/NeuN ratio is decreased in the DG of mice treated with rapamycin. **a** Representative images of SOX2 and NeuN immunofluorescence in the mouse DG. Scale, 200 µm. **b** Magnified images of SOX2 and NeuN immunofluorescence in the mouse DG. Scale, 20 µm. **c** The SOX2/NeuN ratio is decreased in mice treated with rapamycin compared with mice treated with control vehicle, *n* = rapamycin 6 (3 mice), control 6 (3 mice), Student’s *t*-test, *p < 0.05.

SOX2 is a member of the family of SOXB1 transcription factors and functions as an NSC marker as well as have an important role in maintaining multipotent NSC^25^. We assayed the SOX2 levels in the DG of our subject mice using immunofluorescence. The SOX2 signal, normalized by the mature neuron marker NeuN, was decreased in the DG of mice treated with rapamycin (Fig. 3), indicating a reduction in NSC proliferation.

### The frequency of adjustment disorder is higher among kidney transplant patients who treated with rapamycin

To next evaluate the effects of rapamycin in human patients who have undergone a KT, we reviewed the electronic medical records from our university-affiliated hospital. From January 2009 to December 2018, a total of 5498 patients underwent a KT at our institution, of which 861 patients received rapamycin (Supplementary Fig. 3). We found no differences in terms of age (*p* = 0.483), gender (*p* = 0.312), and the glomerular filtration rate (GFR) (*p* = 0.582) between the rapamycin and non-rapamycin (*n* = 4637) group (Table 1). The prevalence of primary kidney disorders (*p* = 0.111) was also comparable between the groups but the DM (*p* = 0.001) was more prevalent in the rapamycin group.

**Table 1:** Rapamycin increases the incidence of psychiatric disorders and psychiatric drug use in KT patients.

	Rapamycin		Non-rapamycin		*t* or *χ*	*p*
	Mean/*n*	SD/%	Mean/*n*	SD/%		
Total KT patients	861		4637			
Age	45.1	16.7	46.2	18.0	0.70	0.483
Male	477	55.40%	2735	58.98%	1.02	0.312
GFR	44.4	16.94	44.2	17.89	0.55	0.582
DM	252	29.27%	981	21.16%	16.66	<0.001
Primary kidney disorders	135	15.68%	618	13.33%	2.54	0.111
Psychiatric disorders						
Depression	16	1.86%	118	2.54%	1.38	0.241
Anxiety^a^	4	0.46%	54	1.16%	3.35	0.067
Adjustment	10	1.16%	18	0.39%	8.44	0.004
Delirium	10	1.16%	74	1.60%	0.89	0.347
Psychotic^a^	2	0.23%	7	0.15%	0.29	0.588
PTSD^a^	0	0	5	0.11%	0.93	0.335
Somatoform^a^	1	0.12%	14	0.30%	0.92	0.338
Insomnia^a^	0	0	4	0.09%	0.74	0.389

*GFR* glomerular filtration rate, *KT* kidney transplantation, *PTSD* post-traumatic disorder.

^a^Fisher’s exact test was done.

Among psychiatric disorders, adjustment disorder was more common in the rapamycin group (*p* = 0.004). There were no differences between the groups in the instances of depression (*p* = 0.241), anxiety (*p* = 0.067), delirium (*p* = 0.347), psychotic disorder (*p* = 0.588), PTSD (*p* = 0.335), somatoform disorder (*p* = 0.338), and insomnia (*p* = 0.389).

## Discussion

We have found in our current study that rapamycin decreases locomotion and sugar consumption in the mouse. Electrophysiological analyses of the DG granule cells in rapamycin-treated mice further revealed decreased excitatory and inhibitory synaptic transmission. Finally, our mouse experiments showed a decreased SOX2/NeuN ratio in the DG in the rapamycin group. In our retrospective analysis of KT patients who received rapamycin, we found an increased frequency of adjustment disorder compared to the transplant recipients that did not receive this drug.

mTOR is known to be related to brain development and its abnormal expression is associated with neuropsychiatric disorders^4,5,26–28^. In addition, mutations in PTEN, an upstream modulator of mTOR, have been associated with autism spectrum disorder and macrocephaly. Tuberous sclerosis complex related genes such as Tsc1 and Tsc2, which are upstream modulators of mTOR, are also associated with autism spectrum disorder and epilepsy^6,7^. Postmortem studies have reported decreases in the NMDAR subunits NR2A and NR2B, the metabotropic glutamatergic receptor 2/3, mTOR and its downstream molecule eIF4B, phospho-eIF4B, and p70S6K in the prefrontal cortex of MDD patients^9,10,29^. Of note in this regard, the NMDAR antagonist ketamine is thought to have acute antidepressant actions through its activation of mTOR signaling^13,16^. On the other hand, calcineurin inhibitors also have deleterious effects on behavior and on brain function^4^. Transplant patients who have received cyclosporine or tacrolimus have been shown to develop psychiatric illnesses including depression, anxiety, and delirium. In our current study series, all of the included KT patients had received a calcineurin inhibitor, thus the effect of calcineurin inhibitor might be canceled out. Overall, our KT study patients who received rapamycin has a higher frequency of adjustment disorder. Moreover, our mouse behavioral tests indicated decreased locomotion and decreased sugar consumption in the rapamycin group, which are thought to be depression-related behaviors.

mTOR is related to synaptic plasticity and synaptic formation^4,27^. mTOR signaling has been established in multiple previous studies as a downstream mechanism of NMDAR-dependent synaptic plasticity^11,14,16,30^. Rapamycin blocks the long-term potentiation (LTP) required for synaptic enhancement and mTOR hyperactivation caused by TSC2 and PTEN mutation in the mouse enhances LTP and epileptic discharge. In our present study, the mice treated with rapamycin showed a decreased frequency of mEPSC and mIPSC in the DG granule cells. The blocking effect of rapamycin on synaptic plasticity was well recapitulated as decreased synaptic transmission.

Newborn neurons and glial cells are differentiated from NSC and proliferate mainly in the subventricular zone and DG in the adult brain^5,28^. Enhanced activation of mTOR potentiates NSC differentiation and proliferation, particularly mTOR complex 1 (mTORC1) activation. mTOR complex 2 activation does not induce NSC differentiation but enhances NSC proliferation. In previous studies, rapamycin was found to suppress proliferation of trophoblasts and gastrulation through the activation of mTORC1, which occurs early in embryonic development^3,7^. Moreover, little has been reported to date about the effects of rapamycin on NSCs^26^. In our present study, SOX2 signaling in the DG NSCs was decreased in mice treated with rapamycin.

CRF and KT have been shown previously to increase the frequency of neuropsychiatric illness^31,32^. Patients with CRF are also at a higher risk of developing certain medical illnesses such as electrolyte imbalance, chronic anemia, cerebral edema, and uremic encephalopathy. CRF patients are also more prone to psychological distress and depression than the general population. Previous studies have reported a 25% prevalence rate of depression in patients with CRF. KT leads to improved and normalized renal function, as well as quality of life, that had been previously impaired by hemodialysis. However, the prevalence of depression in KT patients is still 25%, which is far higher than the general population. Thus, neuropsychiatric illness should be carefully assessed and treated in CRF and KT patients.

The KT patients who received rapamycin in our present study series also had a higher incidence of DM than the non-rapamycin controls. The side effects of rapamycin include lower hyperglycemia, lower nephrotoxicity, less hypertension, and less neoplastic potential compared to calcineurin inhibitor use^26^. Hence, it is possible that more DM patients had been prescribed with rapamycin due to the lower hyperglycemic side effects.

Our present analysis only involved KT patients from our university-affiliated hospital in South Korea. However, the number of patients included in our current study was sufficient to analyze psychiatric illness and medication use with statistical power. Age, gender, GFR, DM, and primary kidney disorders were investigated to assess the possible effects of confounding demographic factors. Our mouse study findings supported the phenomena observed in the patient study. The case–control approaches using rapamycin and control vehicle enabled us to compare the effects of rapamycin and exclude other factors. The comprehensive animal model approach we used, including behavioral, electrophysiological, and immunofluorescence analyses, enhanced the explanatory power of our current investigation.

Our current analysis had some noteworthy limitations however. We used the diagnostic and prescription information from the recorded patient data without any chart reviews. Thus, we could not evaluate the causal relationship of rapamycin prescriptions on neuropsychiatric illnesses from the patient data. Furthermore, not all medical conditions that can lead to a neuropsychiatric illness were investigated in our study series. In the mouse experiments, it was sufficient to evaluate differences in body weight, locomotion, and hedonic behaviors using the moderate number of animals that were included in our behavioral experiments. If a larger number of mice were used in future studies, other behaviors might well be detected such as the anxiety-related disorders reported previously to be induced by rapamycin^33^. In addition, we measured electrophysiology of only granule cells of DG, but not hippocampus neurons. In general, electrophysiological measurements of hippocampus in adult mice are difficult^34^ and electrophysiological measurements of hippocampus in adult mice were not possible under the experimental condition used in the present study. Future work will be needed to provide more extensive results if patch clamps are used using experimental conditions that can be used not only in DG but also in hippocampus in adult mice. Lastly, an EdU or BrdU study with NSCs will help to validate the SOX2 results, but we did not have the resources or experience to do these experiments in our present study.

In conclusion, rapamycin has a promoting impact on neuropsychiatric illness by reducing synaptic transmission and NSC proliferation through the inhibition of mTOR. Clinicians should therefore carefully consider the possibility of neuropsychiatric side effects before or during the administration of rapamycin.

## Supplementary information

Supplementary methods, figures, and table
