# Supplementary material for: Rapamycin increases the incidence of neuropsychiatric illness in kidney transplant patients through the suppression of neural stem cells
Source: Transl Psychiatry. 2020 May 18;10:156. doi: 10.1038/s41398-020-0838-2 (PMC7235015; doi:10.1038/s41398-020-0838-2)
Supplement: Supplementary file 1 — Supplementary methods, figures, and table [file 41398_2020_838_MOESM1_ESM.docx]

**Rapamycin Increases the Incidence of Neuropsychiatric Illness in Kidney Transplant Patients Through the Suppression of Neural Stem Cells**

Yangsik Kim^1,2,3,4,*^, Jung Sun Lee^1^, Yeon Ho Joo^1,*^

^1^Department of Psychiatry, Asan Medical Center, University of Ulsan College of Medicine, Seoul, South Korea; ^2^Grauduate School of Medical Science and Engineering, Korea Advanced Institute of Science and Technology, Daejeon, Korea; ^3^Center for Synaptic Brain Dysfunction, Institute for Basic Science, Daejeon, South Korea; ^4^Mental Health Research Institute, National Center for Mental Health, Seoul, South Korea.

*Correspondence: Yangsik Kim [medicusys@gmail.com](mailto:medicusys@gmail.com) ;

Yeon Ho Joo [jooyh@amc.seoul.kr](mailto:jooyeonho@gmail.com)

**Supplementary Methods and Materials**

**Animal behavior test**

**Open-field test**

Mice were placed in the center region of an open-field box (40 x 40 x 40 cm), and locomotor activity in the open field arena was measured for 60 minutes. Behavioral tests were recorded as video files (avi format) and analyzed using Ethovision XT 12 (Noldus, The Netherlands).

**Elevated plus-maze test**

An elevated-plus maze made of gray acrylic with four arms, each 30-cm long and 5-cm wide, positioned 75 cm above the ground, was used for measuring anxiety-like behavior ^1^. Light conditions in the closed arms were ~0 lux. The test was initiated by placing the mouse in the center of the maze at the junction of the four arms and then allowing the mouse to freely explore the maze for 10 minutes.

**Three-chamber social interaction test**

A three-chambered social-interaction test was performed as described previously ^2, 3^. The apparatus had the following dimensions; W 60 x H 40 x D 20 cm for the whole apparatus, and W 20 x H 20 x D 20 cm for each chamber. The side chambers contained an aluminium grid with a curved face to confine the mouse/object. The assay consisted of three sessions. During the first 10-minute session, a subject mouse was allowed to freely explore all three chambers for habituation. Then the mouse was confined briefly in the center chamber, while a novel object and a WT stranger mouse, stranger 1, were placed in the side chambers behind the aluminum grid in a random manner to minimize the influences of side bias.

All stranger mice were age-matched males and were habituated to the side chambers in advance during the previous day for 30 min. The positions of the object and stranger mouse were alternated between tests to minimize the influences of side preferences.

**Sucrose preference test**

Sucrose preference tests were performed as described previously ^4^. Briefly, mice were housed individually throughout the experiment for four days, followed by one-day habituation to a bottle containing 2% sucrose. Each cage was given two bottles, one containing water and the other containing the 2% sucrose solution, located in two separate places. Mice were allowed to drink freely with a choice between the plain water and sucrose water bottles. Bottles were weighed before testing and at 72 hours after the test. The sucrose preference was defined as the ratio of the amount of consumed sucrose water over the total amount of consumed sucrose water plus plain water.

**Prepulse inhibition test**

For the prepulse inhibition (PPI) test, each mouse was placed on the grid in the chamber, and startle responses were recorded as previously described (San Diego Instruments, San Diego, CA) ^5^. A 65-dB level of background noise, a 120-dB startle stimulus, and various prepulse stimuli (70, 75, 80, 85, and 90 dB) were delivered to the subject mice in a pseudorandom manner. The delay between the prepulse and startle stimulus was 200 msec. The PPI was calculated as follows: 100 – ((mean pre-pulse response/mean pulse response) x 100).

**Fear conditioning**

Fear conditioning tests were performed for 2 days (Coulbourn Instruments, MA). On the first day of testing, the mouse was placed in the center of the chamber and given a foot shock of 0.5mA for 1s for fear-conditioning. At 24 hours after fear learning, the mouse was placed in the same chamber without a foot shock for 5 minutes to measure contextual fear retrieval.

**Electrophysiology: whole-cell recordings**

For electrophysiological assessments, coronal slices were prepared for ACC, MDT, and IC using a vibratome (VT1200S, Leica, Germany) in ice-cold dissection buffer (in mM: 212 sucrose, 25 NaHCO_3_, 5 KCl, 1.25 NaH_2_PO_4_, 10 D-glucose, 2 sodium pyruvate, 1.2 sodium ascorbate, 3.5 MgCl_2_, 0.5 CaCl_2_ bubbled with 95% O2/ 5% CO_2_). The slices were recovered at 32ºC in normal artificial cerebrospinal fluid (ACSF; in mM: 125 NaCl, 25 NaHCO_3_, 2.5 KCl, 1.25 NaH_2_PO_4_, 10 D-glucose, 1.3 MgCl_2_, 2.5 CaCl_2_) and thereafter maintained at room temperature. Cells were visualized using infrared differential interference contrast video microscopy (Olympus, BX50XI, Japan). Whole-cell current-clamp recordings were made using a MultiClamp 700B amplifier (Molecular Devices, CA).

For voltage-clamp recordings, recording pipettes (3–5 MΩ) were filled with a solution containing (in mM) 120 CsMeSO_4_, 15 CsCl, 10 TEA-Cl, 8 NaCl, 10 HEPES, 0.25 EGTA, 5 QX-314, 4 MgATP, and 0.3 NaGTP, pH 7.25–7.35 (280–300 mOsm) ^6^. Signals were filtered at 2 kHz and digitized at 10 kHz. mEPSC values were recorded in the presence of AP5 (50 μM) and tetrodotoxin (1 μM) at the holding potential of -70 mV. mIPSC values were recorded at the holding potential of 0 mV, as described previously ^7^.

**Immunoblotting and Immunofluorescence**

For immunoblotting experiments, a fresh mouse brain was homogenized in ice-cold lysis buffer containing 320 mM sucrose, 10 mM HEPES pH 7.4, 5 mM EDTA, and protease inhibitors. For immunofluorescence experiments, isoflurane-anesthetized mice were transcardially perfused with 4% paraformaldehyde in phosphate-buffered saline (PFA/PBS), followed by brain removal and incubation in 4% PFA/PBS for 24 hours for fixation. Fixed brains were sectioned (100 μm) using a vibratome (VT1200S; Leica, Germany) and subjected to immunofluorescence staining for SOX2 and NeuN.

The following antibodies were purchased commercially: SOX2 antibody (1:1000, Cell Signaling, rabbit, #9234), 𝛂-tubulin antibody (1:10000, Sigma, mouse, T9026), SOX2 antibody (1:1000, R&D systems, mouse, MAB2018), and NeuN antibody (1:1000, Cell Signaling, rabbit, 24307S). For immunoblotting, secondary antibodies against p-S6K and 𝛂-tubulin antibodies were donkey anti-rabbit antibody with 800 nm detection (LiCor, 1:10000) and donkey anti-mouse antibody with HRP (Jackson, 1:10000), respectively. For immunofluorescence, the secondary antibodies for SOX2 and NeuN antibodies were donkey anti-mouse antibody with Alexa 594 (Jackson, 1:1000) and donkey anti-rabbit antibody with Alexa 405 (Jackson, 1:1000), respectively.

**References**

1. Walf AA, Frye CA. The use of the elevated plus maze as an assay of anxiety-related behavior in rodents. *Nature protocols* 2007; **2**(2)**:** 322-328.

2. Silverman JL, Yang M, Lord C, Crawley JN. Behavioural phenotyping assays for mouse models of autism. *Nature reviews Neuroscience* 2010; **11**(7)**:** 490-502.

3. Moy SS *et al.* Sociability and preference for social novelty in five inbred strains: an approach to assess autistic-like behavior in mice. *Genes, brain, and behavior* 2004; **3**(5)**:** 287-302.

4. Seo JS, Zhong P, Liu A, Yan Z, Greengard P. Elevation of p11 in lateral habenula mediates depression-like behavior. *Molecular psychiatry* 2018; **23**(5)**:** 1113-1119.

5. Wells MF, Wimmer RD, Schmitt LI, Feng G, Halassa MM. Thalamic reticular impairment underlies attention deficit in Ptchd1 Y/− mice. *Nature* 2016; **532**(7597)**:** 58.

6. Rothwell PE *et al.* Autism-associated neuroligin-3 mutations commonly impair striatal circuits to boost repetitive behaviors. *Cell* 2014; **158**(1)**:** 198-212.

7. Liang J et al. Conditional neuroligin-2 knockout in adult medial prefrontal cortex links chronic changes in synaptic inhibition to cognitive impairments. *Molecular psychiatry* 2015; **20**(7)**:** 850-859.


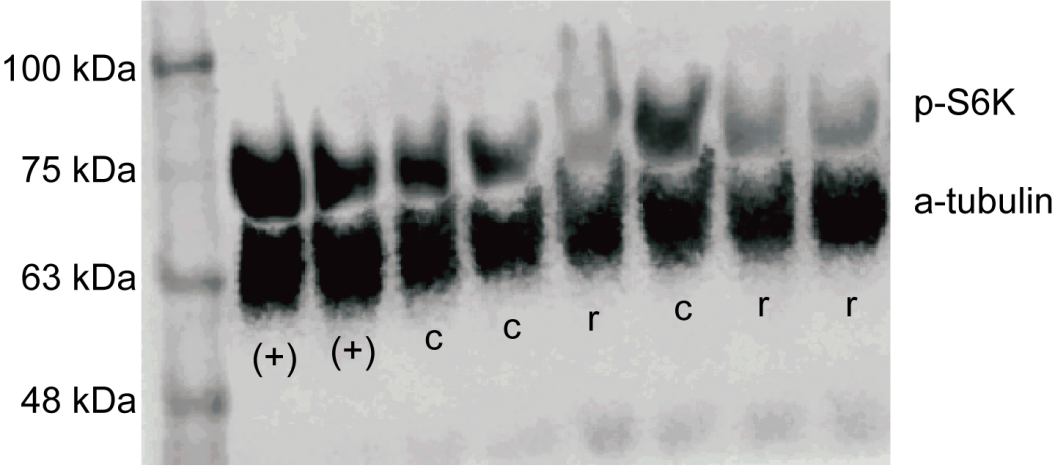


**Supplementary Figure 1. Decreased p-S6K expression in mice treated with rapamycin**

Decreases in the expression of p-S6K in mice treated with rapamycin (12 weeks) were revealed by immunoblotting analysis of whole-brain lysates with p-S6K antibodies. r, rapamycin, c, control vehicle.


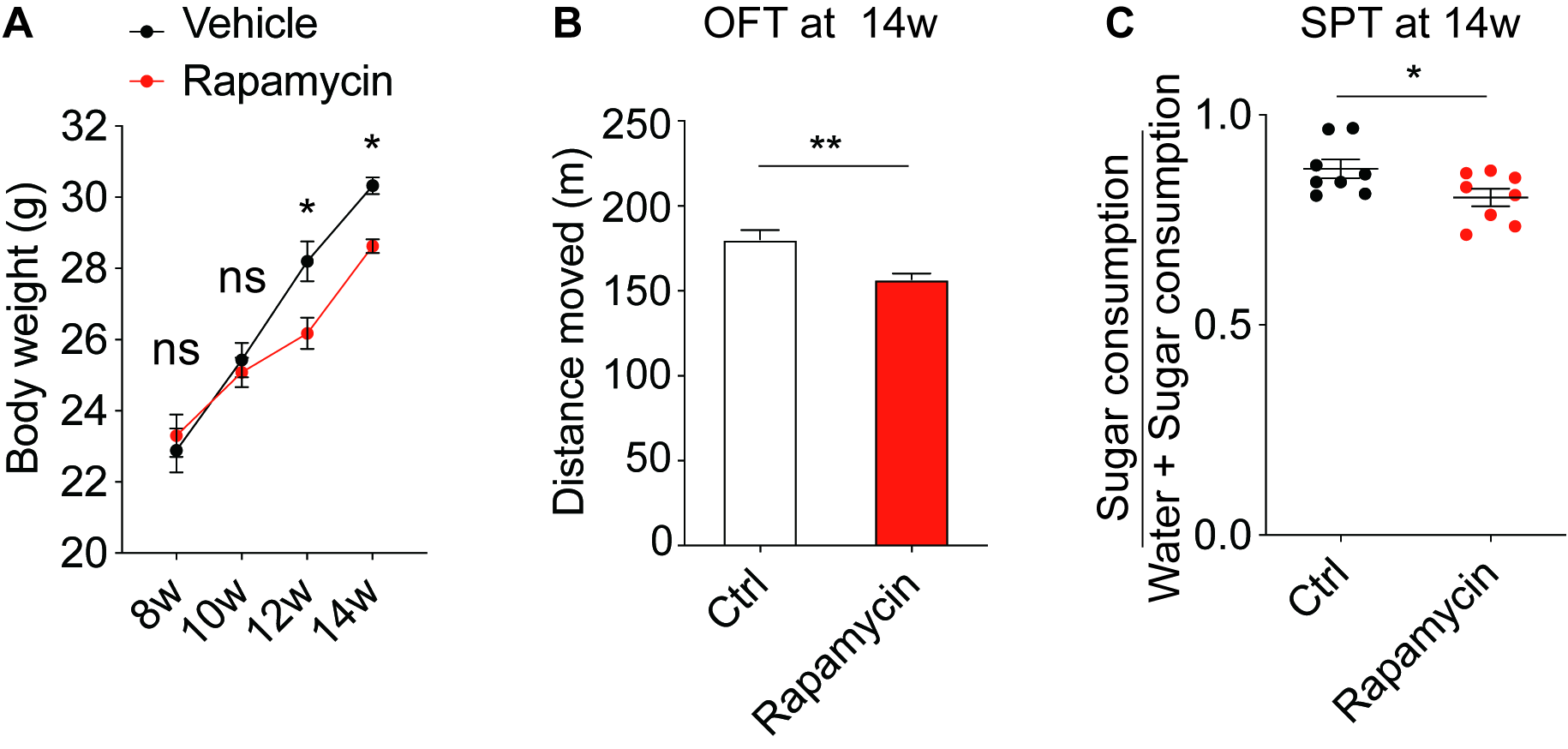


**Supplementary Figure 2. Mice with discontinued rapamycin for 2 weeks exhibited a depression-like behavior**

(A) After stopping rapamycin for 2 weeks, mice showed weight loss compared to animals treated with the control vehicle, n= rapamycin 8, control 8, two-way ANOVA. Bonferroni’s post hoc. (B) Mice treated with rapamycin showed decreased locomotion in an open field test (OFT), n= rapamycin 8, control 8, Student’s t-test. (C) Mice treated with rapamycin had a decreased sugar consumption in a sugar preference test (SPT), n= rapamycin 8, control 8, Student’s t-test. ns, not significant, *p<0.05, **p<0.01.


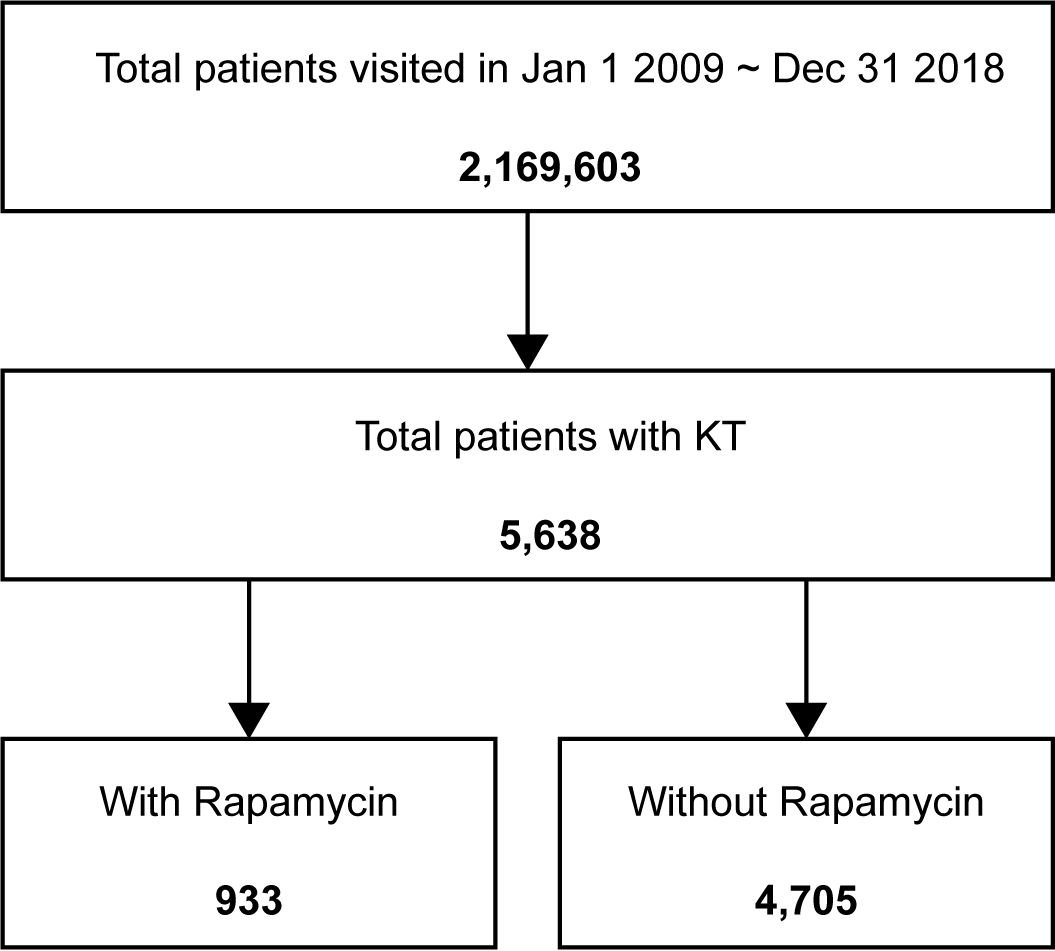


**Supplementary Figure 3. Enrollment process of KT patients**

| **Supplementary Table 1. Statistical details** | | | | | | | | | | | |
| --- | --- | --- | --- | --- | --- | --- | --- | --- | --- | --- | --- |
| Figure | Experiment | Number  of subjects | Descriptive,  mean | Descriptive,  SEM | Statistics | | | | | | |
| 2 | Body Weight | ctrl: 7 | 25.8 | 1.793 | One-way  ANOVA | ANOVA table | SS | DF | MS | F (DFn, DFd) | P value |
|  |  | rapamycin: 8 | 24.79 | 0.8282 | post-hoc:  Bonferonni's | Interaction | 24.1 | 2 | 12.05 | F (2, 39) = 3.497 | P=0.0401 |
|  |  |  |  |  |  | time | 150.7 | 2 | 75.35 | F (2, 39) = 21.87 | P<0.0001 |
|  |  |  |  |  |  | genotype | 11.58 | 1 | 11.58 | F (1, 39) = 3.361 | P=0.0744 |
|  | OFT | ctrl: 7 | 178.89 | 12.25 | Unpaired t test |  |  |  |  |  |  |
|  |  | rapamycin: 8 | 137.11 | 9.4 | P value | 0.0167 | t=2.744, df=13 |  |  |  |  |
|  | SPT | ctrl: 7 | 0.8612 | 0.01836 | Unpaired t test |  |  |  |  |  |  |
|  |  | rapamycin: 8 | 0.8012 | 0.01939 | P value | 0.0442 | t=2.228, df=13 |  |  |  |  |
|  | EPM | ctrl: 7 | 233.2 | 184.9 | Two-way  ANOVA | ANOVA table | SS | DF | MS | F (DFn, DFd) | P value |
|  |  | rapamycin: 8 | 205.7 | 141.1 | post-hoc:  Bonferonni's | Interaction | 13472 | 1 | 13472 | F (1, 24) = 3.588 | P=0.0703 |
|  |  |  |  |  |  | arms | 743893 | 1 | 743893 | F (1, 24) = 198.1 | P<0.0001 |
|  |  |  |  |  |  | genotype | 5281 | 1 | 5281 | F (1, 24) = 1.407 | P=0.2472 |
|  | 3CT | ctrl: 7 | 79.58 | 22.07 | Two-way  ANOVA | ANOVA table | SS | DF | MS | F (DFn, DFd) | P value |
|  |  | rapamycin: 8 | 66.64 | 21.57 | post-hoc:  Bonferonni's | Interaction | 1.924 | 1 | 1.924 | F (1, 26) = 0.002224 | P=0.9627 |
|  |  |  |  |  |  | S-O | 14220 | 1 | 14220 | F (1, 26) = 16.44 | P=0.0004 |
|  |  |  |  |  |  | genotype | 1249 | 1 | 1249 | F (1, 26) = 1.445 | P=0.2402 |
|  | PPI | ctrl: 7 | 38.1 | 5.75 | Two-way  ANOVA | ANOVA table | SS | DF | MS | F (DFn, DFd) | P value |
|  |  | rapamycin: 8 | 36.23 | 7.423 | post-hoc:  Bonferonni's | Interaction | 236.3 | 4 | 59.08 | F (4, 70) = 0.1972 | P=0.9390 |
|  |  |  |  |  |  | dB | 13870 | 4 | 3467 | F (4, 70) = 11.57 | P<0.0001 |
|  |  |  |  |  |  | genotype | 70.33 | 1 | 70.33 | F (1, 70) = 0.2348 | P=0.6295 |
|  | Fear habituation | ctrl: 7 | 16.01 | 7.443 | Two-way  ANOVA | ANOVA table | SS | DF | MS | F (DFn, DFd) | P value |
|  |  | rapamycin: 8 | 14.15 | 6.717 | post-hoc:  Bonferonni's | Interaction | 178.9 | 5 | 35.79 | F (5, 78) = 0.2924 | P=0.9158 |
|  |  |  |  |  |  | time | 22339 | 5 | 4468 | F (5, 78) = 36.50 | P<0.0001 |
|  |  |  |  |  |  | genotype | 77.46 | 1 | 77.46 | F (1, 78) = 0.6328 | P=0.4287 |
|  | Contextual fear learning | ctrl: 7 | 27.18 | 4.264 | Unpaired t test |  |  |  |  |  |  |
|  |  | rapamycin: 8 | 28.81 | 2.944 | P value | 0.7534 | t=0.3209, df=13 |  |  |  |  |
| 3 | mEPSC  frequency | ctrl: 15 | 0.2283 | 0.02298 | Mann-Whitney |  |  |  |  |  |  |
|  |  | rapamycin: 10 | 0.05482 | 0.02779 | P value | 0.0003 | U=14 |  |  |  |  |
|  | mEPSC  amplitude | ctrl: 15 | -21.67 | 0.7412 | Unpaired t test |  |  |  |  |  |  |
|  |  | rapamycin: 10 | -22.89 | 1.318 | P value | 0.3934 | t=0.8680, df=23 |  |  |  |  |
|  | mIPSC  frequency | ctrl: 15 | 0.3094 | 0.05206 | Mann-Whitney |  |  |  |  |  |  |
|  |  | rapamycin: 11 | 0.175 | 0.05465 | P value | 0.0168 | U=37 |  |  |  |  |
|  | mIPSC  amplitude | ctrl: 15 | 22.17 | 0.5398 | Unpaired t test |  |  |  |  |  |  |
|  |  | rapamycin: 11 | 23.36 | 0.4779 | P value | 0.1276 | t=1.578, df=24 |  |  |  |  |
| 4 | SOX2/NeuN  ratio | ctrl: 6 | 0.4434 | 0.02659 | Unpaired t test |  |  |  |  |  |  |
|  |  | rapamycin: 6 | 0.3388 | 0.02491 | P value | 0.0166 | t=2.871, df=10 |  |  |  |  |
| S2 | Body Weight | ctrl: 8 | 26.71 | 1.621 | One-way  ANOVA | ANOVA table | SS | DF | MS | F (DFn, DFd) | P value |
|  |  | rapamycin: 8 | 25.79 | 1.114 | post-hoc:  Bonferonni's | Interaction | 15.72 | 3 | 5.240 | F (3, 56) = 3.021 | P=0.0372 |
|  |  |  |  |  |  | time | 355.9 | 3 | 118.6 | F (3, 56) = 68.38 | P<0.0001 |
|  |  |  |  |  |  | genotype | 13.41 | 1 | 13.41 | F (1, 56) = 7.733 | P=0.0074 |
|  | OFT | ctrl: 8 | 180.15 | 5.48 | Unpaired t test |  |  |  |  |  |  |
|  |  | rapamycin: 8 | 156.88 | 3.36 | P value | 0.0028 | t=3.621, df=14 |  |  |  |  |
|  | SPT | ctrl: 8 | 0.8716 | 0.02237 | Unpaired t test |  |  |  |  |  |  |
|  |  | rapamycin: 8 | 0.8034 | 0.02086 | P value | 0.0425 | t=2.231, df=14 |  |  |  |  |

SEM, standard error of the mean; ANOVA, analysis of variation; SS, sum of squares ;DF, degrees of freedom; MS, mean squares.
